# The impact of the COVID-19 pandemic on the performance of the Rapid Access Lung Cancer Clinic

**DOI:** 10.1007/s11845-024-03749-8

**Published:** 2024-07-22

**Authors:** Mohammad J. Ghassemi-Rad, Colum Dennehy, Noreen Lyons, Michael T. Henry, Marcus P. Kennedy, Éilis J. O’Reilly, Roisin M. Connolly

**Affiliations:** 1 School of Medicine, College of Medicine and Health, University College Cork, Cork, Republic of Ireland; 2 Medical Oncology, Cork University Hospital, Wilton, Republic of Ireland; 3 Rapid Access Lung Cancer Clinic, Department of Respiratory Medicine, Cork University Hospital, Wilton, Republic of Ireland; 4 School of Public Health, College of Medicine and Health, University College Cork, Cork, Republic of Ireland; 5 Cancer Research @ UCC, College of Medicine and Health, University College Cork, Western Gateway Building, 4.110, Western Road, Cork, Republic of Ireland

**Keywords:** COVID-19, Lung cancer, Pandemic, Rapid access lung cancer clinic

## Abstract

**Background:**

The Rapid Access Lung Cancer Clinic (RALC) experienced fewer referrals during the COVID-19 pandemic in Ireland.

**Aims:**

Our aim was to determine the impact of the pandemic on the key performance indicators (KPIs) of the Cork University Hospital (CUH) RALC, using a retrospective chart review of the referrals and attendances.

**Methods:**

The medical charts of patients referred to CUH-RALC from 03/2019 to 02/2020 (period I), and from 03/2020 to 02/2021 (period II) were reviewed. Performance of the RALC was determined based on average wait time from referral to 1] acquisition of the first CT scan, 2] consultation, and 3] receiving a cancer diagnosis, and compared between periods I and II.

**Results:**

Average monthly referrals (57.3 vs 42.1, *p* = 0.0078) and RALC reviews (24.3 vs 22, *p* = 0.0310) were lower in period II compared to period I. However, no difference was seen in the length of time from referral to review at RALC or time to receive cancer diagnosis. There were shorter wait times from referral to CT scan (11.2 vs. 8.7 days, *p* = 0.0011) and to surgery (109.0 vs 79.3 days, *p* = 0.0236) in period II.

**Conclusions:**

The COVID-19 pandemic had minimal impact on the performance of RALC at our institution. Fewer referrals to RALC in period II may relate to hesitancy in attending general practitioner (GP) and/or GPs raising the thresholds for referrals to RALC during the early lockdown period of the pandemic. A national evaluation will be required to fully determine the impact of this pandemic on lung cancer in Ireland.

## Introduction

Lung cancer is among the most common cancers diagnosed in Ireland and indeed globally [1, 2]. With more than 2 million cases worldwide and 2700 cases in Ireland diagnosed annually, lung cancer is also the leading cause of cancer-related deaths [1, 2]. Despite many therapeutic options, most patients succumb to the disease with only 20% of patients surviving up to five years in Ireland [2]. The morbidity and mortality associated with lung cancer is primarily related to the late stage of presentation and diagnosis. Most lung cancers present at stages III and IV when cancer has already progressed and metastasized [2, 3].

To reduce lung cancer mortality in Ireland, Rapid Access Lung Cancer Clinics (RALC) were established in 2009 by the National Cancer Control Programme (NCCP) at eight cancer centres for early assessment of individuals with clinical suspicion of lung cancer [3]. Most patients with lung cancer, who have an abnormal chest radiograph or show warning signs and symptoms such as haemoptysis, unexpected weight loss, or persistent new cough for over three months, are referred to RALC for consultation by respiratory physicians with a special interest in lung cancer. In accordance with the NCCP guidelines, patients must be seen at the RALC for clinical assessment within 10 working days (12 calendar days) of receipt of referral [3, 4]. Key Performance Indicators (KPIs) have been developed for RALC to ensure patients are seen in the clinic, discussed at multi-disciplinary team (MDT) meetings, and receive surgery or systemic therapy within a recommended timeframe; thereby improving the delivery of care to these patients [4, 5]. Meanwhile, delays in referrals, obtaining the diagnosis, and starting treatment can negatively impact survival outcomes in patients with lung cancer [6, 7].

In 2020 and 2021 healthcare services were required to primarily focus their resources and efforts on combatting the COVID-19 pandemic. For instance, lower hospital admissions and higher mortality rates were observed with cardiovascular diseases during periods of peak transmission of the pandemic in the United Kingdom (UK) and Italy [8, 9]. In the UK, patients with a cancer diagnosis were not only vulnerable to worse outcome following COVID-19 infection [10, 11], but they also experienced potential delays in consultations, receipt of diagnostic imaging services, and start of treatment during the pandemic [12, 13]. Many governing bodies, including the European Society for Medical Oncology (ESMO), advised that all patients with a clinical suspicion of lung malignancy should have priority access to diagnostic imaging and surgeries throughout the pandemic and delays in treatment should not exceed eight weeks [14].

In Ireland, of the eight total RALC at our designated cancer centres, two have reported reduced activity leading to fewer referrals and greater delays in diagnosis during the early months of the pandemic [15, 16]. Internationally, suspension of screening programs, lockdowns, fear of infection contributing to missed referrals or follow-up care, and altered treatment pathways such as suspension of some clinical trials are some of the important implications of COVID-19 pandemic for cancer patients [12].

The objective of our study was to investigate the impact of the COVID-19 pandemic on the KPIs of the RALC at Cork University Hospital (CUH), a large university teaching hospital in Southern Ireland, using a retrospective chart review of the 2019–2021 referrals and attendances. We hypothesized that the COVID-19 pandemic resulted in a significant delay in obtaining diagnostic imaging, clinical review, diagnosis, MDT meeting, and start of therapy via the RALC pathway potentially leading to poor survival outcome for the patients.

## Methods

### Study design

This retrospective quantitative study commenced following ethical approval from Clinical Research Ethics Committee (CREC) of the Cork teaching hospitals. The Irish NCCP RALC pathway streamlines and standardizes the referral of patients suspected of having lung cancer (i.e., signs and symptoms and/or abnormality on chest X-ray with a differential diagnosis of lung cancer) by general practitioner (GP), other health care providers or emergency services [17]. Upon receipt of the referral, it is triaged by a respiratory consultant with a special interest in lung cancer. Patients may be discharged or triaged to another pathway depending on the appropriateness of the referral. However, most patients undergo a computed tomography (CT) thorax and upper abdomen with contrast as the gold standard for lung cancer detection (post-referral CT), although some patients have CT scans prior to referral (pre-referral CT). The results of the CT scan are reported by consultant radiologists with interest in respiratory disease and assessed by respiratory consultant with a special interest in lung cancer. Patients are then triaged to either RALC, virtual nodule (VN) clinic, respiratory clinic, or discharged.

Our study design selected two timeframes to compare a number of outcomes and adherence to national KPIs for patients attending CUH-RALC. The two comparison periods were defined as March 2019 to February 2020 (period I), which is one year prior to the start of the pandemic, and March 2020 to February 2021 (period II) as the first year of the pandemic in Ireland. Outcomes analysed included average wait times for a clinical consultation at RALC, acquiring a diagnostic CT scan, receiving a diagnosis, discussion at MDT meeting, and starting treatment. Furthermore, the number of referrals, RALC consultations (reviews), lung cancer diagnoses, and stages of lung cancers were also be compared.

### Data collection

Electronic health records of all patients referred to the CUH-RALC from January 2019 to February 2021 were reviewed. Patient’s demographics including; age, gender, as well as their date of referral to RALC, date of first CT scan, date of initial review at RALC, outcome of RALC review, date of cancer diagnosis, type of lung cancer, cancer stage at diagnosis, date of first thoracic oncology MDT discussion, date of thoracic surgery or systemic anti-cancer therapy (SACT) initiation, and date of death, were collected and kept in a password-protected database. Patient’s demographic data are presented as monthly average referrals and reviews to RALC for periods I and II.

To analyse progression-free survival (PFS) and overall survival (OS) for patients diagnosed with lung cancer, the length of time (in months) from diagnosis to recurrence / progression and death were used. The follow-up period at the time of data analysis was seven months for period II. Therefore, to eliminate longer follow-up for period I, the follow-up for both periods was set at seven months after the end of each period, September 2020 for period I and September 2021 for period II. Analysis of PFS and OS was also to be compared for lung cancer subgroups separately including the stages I-III and stage IV cohorts.

### Statistical analysis

Data was analysed and graphs were generated with GraphPad Prism (Version 6.07, GraphPad Software, Inc., La Jolla, CA). A paired t-test with Wilcoxon matched-pairs was used for comparing the number of referral and RALC reviews in period I and II. Average time to acquire the first CT scan, consultation at RALC, and receiving a histological diagnosis of cancer were calculated using the date of referral and compared between periods I and II using an unpaired t-test (Mann–Whitney). Proportions and the stages of lung cancers were compared in periods I and II using a Chi-squared test. PFS and OS data were plotted on a survival curve and analysed using a Gehan-Breslow-Wilcoxon test. Results were considered significant at p < 0.05.

## Results

### Demographics

Of the 1192 medical charts reviewed; 687 patients were referred to RALC in period I and 505 patients in period II (Table 1); indicating a 26.5% reduction in the number of referrals during the first year of the pandemic when compared to the pre-pandemic year. Average age, gender, and the county of origin of patients referred to RALC during these two periods have largely remained the same (Table 1).

**Table 1:** Demographics, Triage and Clinic Outcome of patients who were referred to CUH RALC

	Period I	Period II	Total
**REFERRALS**	**Total *N* = 687**	**Total *N* = 505**	**Total *N* = 1192**
Sex, Male/Female (%)	375/312 (55/45)	267/238 (53/47)	642/550 (54/46)
Age in years: mean (median) [range]	64.09 (65) [22–98]	65.24 (68) [26–90]	64.58 (66) [22–98]
Age 10%-90% centile	45-80	45.6 – 80.4	45.3—80
**TRIAGE (%)**	**Total *N* = 687**	**Total *N* = 505**	**Total *N* = 1192**
Pre-referral CT*	283 (41)	246 (49)	529 (44)
Post-referral CT	348 (51)	233 (46)	581 (49)
Discharge	56 (8)	26 (5)	82 (7)
**RALC REVIEWS**^**#**^ **(%)**	**Total *N* = 365 (53)**	**Total *N* = 286 (57)**	**Total *N* = 651 (55)**
Lung cancer work-up	124 (34)	109 (38)	233 (36)
Other cancers work-up	27 (7)	21 (7)	48 (7)
Respiratory clinic	23 (6)	16 (6)	39 (6)
VN clinic	49 (14)	34 (12)	83 (13)
RALC follow-up	92 (25)	66 (23)	158 (24)
Discharge	50 (14)	40 (14)	90 (14)

*Some patients already have a CT-scan done before being referred to RALC

#Outcome of consultation at RALC

### Monthly referrals and reviews at CUH-RALC

**Fig. 1:**
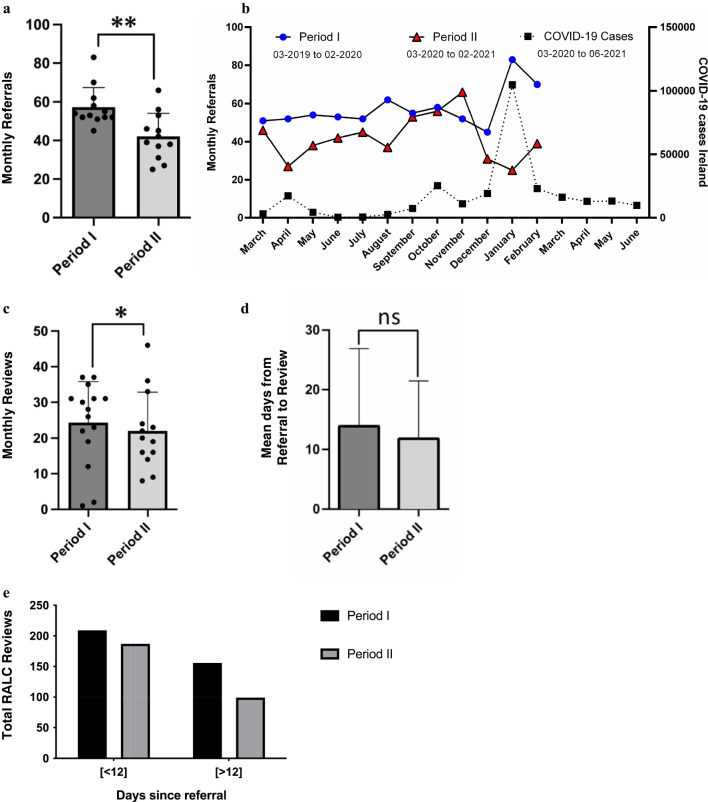
Monthly referral and reviews at CUH-RALC. (**a**) Each dot represents total number of referrals to CUH-RALC for each month. Bar graph represents mean value for monthly referral ± standard deviation (SD) (*p* = 0.0078). (**b**) Monthly referrals to CUH-RALC before and during the pandemic with the corresponding monthly COVID-19 cases in Ireland obtained from OurWorldInData website [18]. (**c**) Each dot represents total number of reviews at CUH-RALC for each month. Some reviews were done outside the respective period but included in the analysis. Bar graph represents mean values for monthly reviews ± SD (*p* = 0.0310). (**d**) Mean for the number of days from referral to review at CUH-RALC ± SD (*p* = 0.1075). (**e**) Occurrence of RALC reviews within the specified time intervals (OR 1.41, *P* = 0.035, 95% CI 1.024 – 1.941). * and ** denotes statistical significance at *p* < 0.05 and *p* < 0.01, respectively, using a Wilcoxon matched pairs test (**a** and **c**), a Mann–Whitney test (**d**), and a chi-squared analysis (**e**). (CUH: Cork University Hospital, RALC: Rapid Access Lung Clinic, OR: Odds ratio, CI: Confidence interval, SD: Standard deviation)

Mean monthly referrals were significantly lower in period II (*n* = 42.0) compared to period I (*n* = 57.3) (*p* = 0.0078, Fig. 1a). Interestingly, the lowest monthly referrals in period II correspond with the COVID-19 peaks in April 2020 and January 2021 in Ireland (Fig. 1b).

Consistent with the reduction in referrals to RALC during period II, there was a significant reduction in the number of monthly patient reviews at the RALC during that period (*p* = 0.0310, Fig. 1c). A total 15 reviews at the RALC for period I referrals were done over 3 months during period II, and 8 of the reviews for period II referrals were done after the end of the period over 1 month. Hence, additional dots on the figure indicate the extra months after the end of each period when RALC reviews were done after the period had ended (Fig. 1c).

Also, no significant difference was seen in the wait times from referral to review at RALC (*p* = 0.1075) between periods I and II (Fig. 1d). Mean wait time was 14 days in period I, and 12 days in period II. However, it was also observed that in period I, 209 out the 365 reviews occurred within 12 calendar days of referral (57%); while in period II 187 out of 286 RALC reviews (65%) happened within 12 calendar days of referral (OR 1.41, *p* = 0.035, 95% CI 1.024 – 1.94, Fig. 1e). This is a noticeable increase in the number of RALC reviews happening within 12 calendar days of referral during period II compared to period I.

### Pre- and post-referral CT scans

**Fig. 2:**
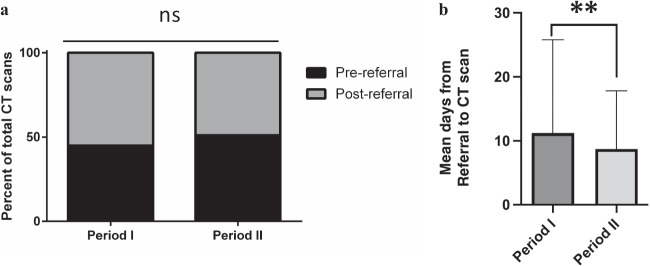
Frequency and wait times for CT scans for attendees at CUH-RALC. (**a**) Percentage of pre-referral and post-referral CT scans during periods I and II at CUH-RALC compared to total number of CT scans for each period. A Chi-squared test was performed (OR 1.27, *p* = 0.39, 95% CI 0.73 – 2.22). (**b**) Average number of days lapsed from referral to CT scans at CUH-RALC during periods I and II ± SD (11.2 vs. 8.7 days, *p* = 0.0011). ** denotes statistical significance at *p* < 0.01 using a Mann–Whitney test

There were 24% fewer CT scans performed in period II compared to period I (631 vs 479, Table 1). This reduction is most evident in post-referral CT scans (33% reduction) compared to pre-referral CT scans (13% reduction) (Table 1). However, both periods were associated with a similar percentage of pre-referral and post-referral CT scans when compared to the total number of CT scans performed in each period (Fig. 2a; 45% and 55% in Period I vs. 51% and 49% in Period II respectively; *p*-value 0.39). The wait times from referral to CT scan in period II was significantly lower compared to period I (*p* = 0.0011, Fig. 2b).

### Frequency and stages of lung cancers

The outcome of RALC reviews with respect to frequencies and stages of lung cancers diagnosed were similar between the two periods (Table 1). A total of 155 and 136 cancers were diagnosed as a result of CUH-RALC consultations during periods I and II, respectively (Table 2). Of these, 82% were lung cancers in each period; with other cancers diagnosed including gastrointestinal, haematologic amongst others. The frequency of all cancers detected did not differ between periods I and II (Table 2).

**Table 2:** Patient and tumour characteristics for lung cancers diagnosed at CUH-RALC during periods I and II

	Period I	Period II	Total	*p*-value
**PATIENT CHARACTERISTICS**	**Total *N* = 155**	**Total *N* = 136**	**Total *N* = 291**	
Sex, Male/Female (%)	90/65 (58/42)	78/58 (57/43)	168/123 (58/42)	0.14
Age in years, mean (median) [Range]	69.34 (71) [37–89]	68.74 (70.50) [38–87]	69.06 (71) [37–89]	0.73
Age 10%-90% centile	56-82	56-80	56-81	
**CANCER TYPES (%)**				0.93
Lung	127 (82)	111 (82)	238 (82)	
GI* Cancer	6 (4)	5 (3.5)	11 (4)	
Haematologic	6 (4)	4 (3)	10 (3)	
Head and Neck	5 (3)	9 (6.5)	14 (5)	
Other^	11(7)	7 (5)	18 (6)	
**STAGE AT INITIAL LUNG CANCER DIAGNOSIS (%)**	**Total *N* = 127**	**Total *N* = 111**	**Total *N* = 238**	0.15
1A	15 (12)	11 (10)	26 (11)	
1B	9 (7)	5 (4)	14 (6)	
2	12 (9)	14 (13)	26 (11)	
3A	10 (8)	17 (15)	27 (11)	
3B	13 (10)	13 (12)	26 (11)	
4	68 (54)	51 (46)	119 (50)	
**HISTOLOGY OF LUNG CANCERS (%)**				0.81
Adenocarcinoma	78 (61)	54 (49)	132 (55)	
Squamous cell	30 (24)	37 (33)	67 (28)	
Large cell carcinoma (not specified)	2 (1.5)	0 (0)	2 (1)	
Small cell	15 (12)	18 (16)	33 (14)	
Other^#^	2 (1.5)	2 (2)	4 (2)	

*GI: Gastro-intestinal

^Other includes Gynaecological, Genito-urinary, Melanoma, Breast

#Other includes Carcinoid and mesothelioma

**Fig. 3:**
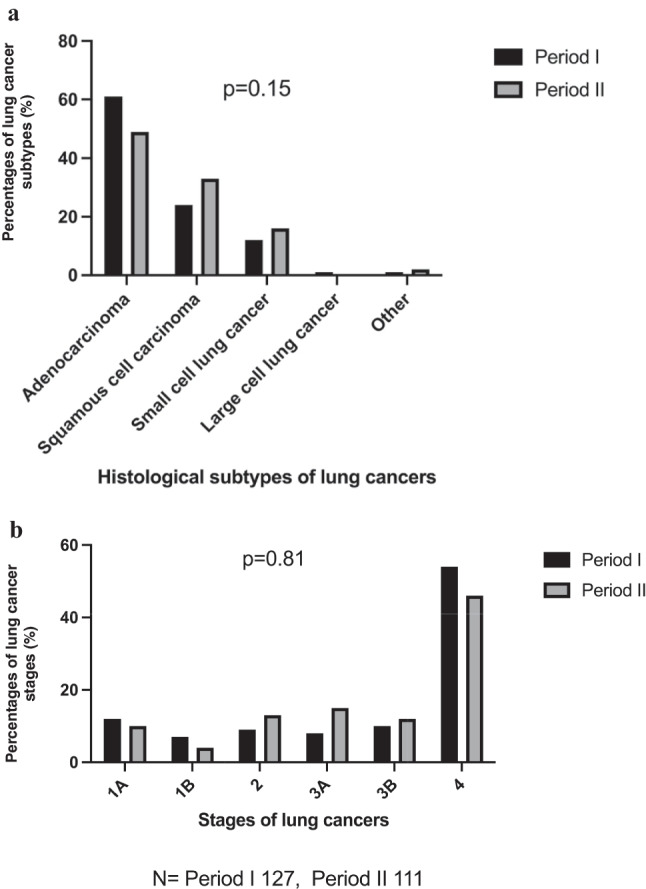
Percentages of histological types and stages of lung cancers diagnosed at CUH-RALC during period I and II. Percentage of lung cancer histology (**a**) and stages at initial diagnosis (**b**) were analysed using a Chi-squared test (a: *p* = 0.15; b: *p* = 0.81)

The majority of lung cancers diagnosed across the two periods were adenocarcinomas (78 [61%] in period I, 54 [49%] in period II), with squamous cell cancers being the second most common (30 [24%] in period I, 37 [33%] in period II) in keeping with international patterns (Table 2). However, comparison of lung cancer subtypes and stages between periods I and II revealed no statistically significant difference (*p* = 0.15, *p* = 0.81, respectively; Fig. 3).

### Diagnosis, management, and survival of patients with lung cancer

**Fig. 4:**
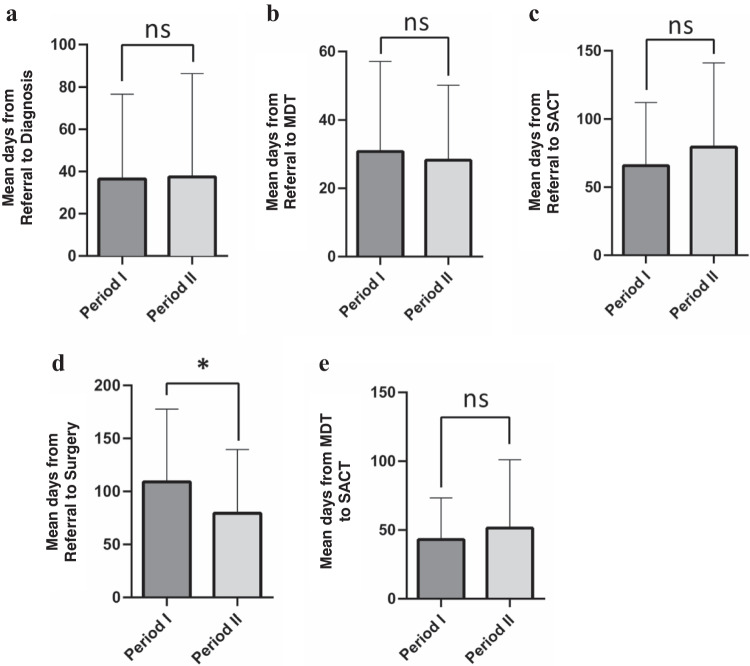
Time to lung cancer diagnosis, MDT meeting, and start of treatment for patients with lung cancer during periods I and II. Bar graphs represent the mean value for number of days lapsed from referral to (**a**) diagnosis (*p* = 0.94), (**b**) MDT (*p* = 0.86), (**c**) SACT (*p* = 0.23), and (**d**) surgery (*p* = 0.023), as well as (**e**) time lapsed from MDT to SACT (*p* = 0.60) ± SD. * denotes statistical significance at *p* < 0.05 using a Mann–Whitney test. (MDT: Multi-Disciplinary Team, SACT: Systemic Anti-cancer Therapy.)

No significant change was observed in wait times from referral to histological diagnosis (mean of 36.5 vs. 37.4 days, median of 27 vs. 28 days, *p* = 0.94), MDT meeting (mean of 30.8 vs. 28.2 days, median of 25 vs. 23 days, *p* = 0.86), or start of SACT (mean of 66 vs. 79 days, median of 57 vs. 64 days, *p* = 0.23) from period I to II (Fig. 4a-c). Similarly, the wait time from MDT to the start of SACT has been similar between the two periods (mean of 43 vs. 51.4 days, median of 37 vs. 38 days, *p* = 0.60) (Fig. 4e). However, patients diagnosed with lung cancer in period II received surgery sooner than patients in period I (mean of 109 vs. 79 days, median of 89 vs 59 days, *p* = 0.023) (Fig. 4d).

**Fig. 5:**
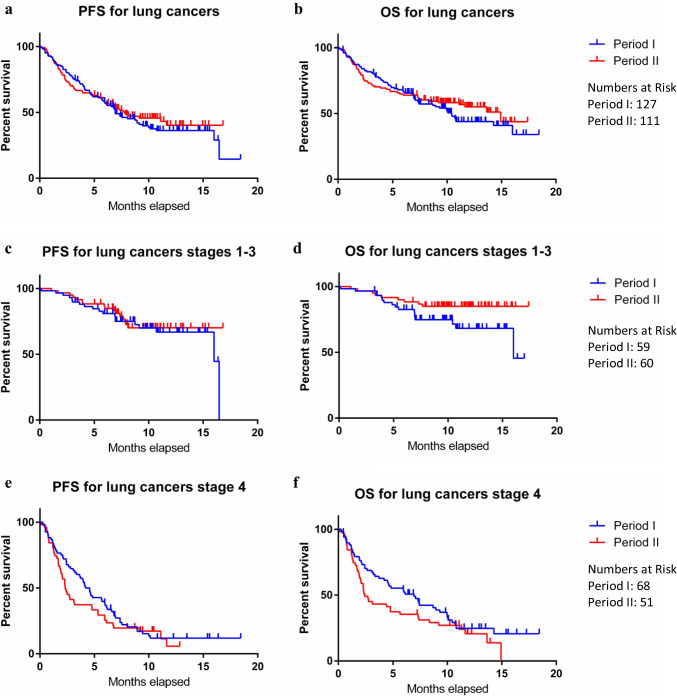
PFS and OS for all lung cancer patients, and stages 1–3 and 4 subgroups, diagnosed at CUH-RALC during periods I and II. PFS and OS were calculated from the diagnosis date until 7 months after the end of each period (September 2020 for period I and September 2021 for period II), hence the x-axis shows 18-month time follow-up. Results were plotted on a survival curve and analysed using a Gehan-Breslow-Wilcoxon test. P-values are as follows: (**a**) 0.95, (**b**) 0.81, (**c**) 0.70, (**d**) 0.12, (**e**) 0.11, (**f**) 0.09. (CUH: Cork University Hospital, RALC: Rapid Access Lung Clinic, PFS: Progression free survival, OS: Overall survival)

Median PFS for patients with lung cancer was 6.9 and 7.8 months, while median OS for patients with lung cancer were 10.4 and 14.9 months for periods I and II, respectively. However, there was no significant difference in PFS and OS for lung cancer patients between periods I and II (PFS *p* = 0.95, OS *p* = 0.81, Fig. 5a-b). Furthermore, no noticeable difference was observed between period I and II regarding the PFS and OS of patients with lung cancer in stages I-III (Fig. 5c-d) and stage IV (Fig. 5e-f) cohorts.

## Discussion

The RALC pathway was established by the NCCP in order to facilitate timely radiological and clinical assessment of individuals with clinical suspicion of having lung cancer in Ireland [3]. Unfortunately, the COVID-19 pandemic had a huge impact on healthcare services across the world, including cancer care [19–21]. Some of the RALC centres in Ireland were not spared [15, 16]. Early reports during the COVID-19 pandemic indicated that Irish RALC centres in Limerick and Beaumont hospitals experienced reduced number of referrals, and increased wait times for patient review at RALC. More importantly, a higher proportion of lung cancers diagnosed were stage IV, when compared to prior years [15, 16]. In contrast, our findings indicate that the COVID-19 pandemic had minimal impact on the performance and the outcome of CUH-RALC within the study timeframe. Although the numbers of referrals and reviews at our centre dropped significantly during the pandemic (period II, Fig. 1), similar to other parts of the country [15, 16], no significant change was noticed in the stages or proportions of lung cancers diagnosed pre- and during the pandemic (Fig. 3). Also, the difference seen on the lung cancer data between periods I and II could be due to the year-to-year variability. However, a significant reduction in referrals to RALC during the pandemic may have been related to fewer visits to the GP in the setting of national lockdowns, or GPs raising the threshold for referrals to the acute hospital setting to avoid patient exposure or over burdening the healthcare system during a time of crisis. Furthermore, the average wait time for RALC review did not change significantly during the pandemic (14 vs. 12 days) but significantly more patients were being seen within 10 working days (12 calendar days) of referral during the pandemic compared to before (OR 1.41, Fig. 1e). These findings suggest that the reduction in referrals during the pandemic period reduced the burden on the RALC in terms of patient volume.

Radiology is one of the key modalities of COVID-19 diagnosis [22]. During the pandemic, the radiology departments had to adapt and prioritise to maintain activity for influx of COVID-19 patients, while continue supporting non-COVID-19 patients such as cancer patients and those in emergency situations. Our results indicate that there has been a noticeable reduction in the total number of CT scans performed at RALC during the COVID-19 pandemic, consistent with reduction in the number of referrals to RALC. Interestingly, the wait times for post-referral CT scans were significantly shorter during the pandemic compared to before, suggesting that the reduction in referrals may have led to increased availability of CT scan slots. Another possibility is that there were less patients undergoing CT imaging for other general indications. In the UK, the beginning of the lockdowns resulted in reduction of referrals from GPs and secondary care to imaging services and the elimination of backlogs allowing the more essential tests to be done, which is consistent with our findings at CUH-RALC [23]. However, the long-term effect of some of the delays regarding imaging in post-COVID-19 era, particularly for cancer patients remains to be seen.

The results of this study indicate that there have been no delays in time to diagnosis, MDT meeting, or start of treatment for lung cancer patients coming through the RALC pathway in one year since the start of the pandemic (Fig. 4). The average wait time from MDT meeting to start of SACT for lung cancer patients was 51.4 days in period II which is not significantly different from the wait time before the pandemic (Fig. 4E). This wait time is consistent with ESMO recommendation of maximum eight weeks between MDT meeting and start of treatment for lung cancer patients during the pandemic [14].

Throughout the pandemic, the respiratory physicians and clinical nurse specialists at RALC also had to look after the influx of additional COVID-19 / pneumonia patients and adapt to the new challenges associated with access to diagnostic imaging services including CT, Positron Emission Tomography, bronchoscopy, and endobronchial ultrasound. However, our results indicate that they continued to prioritise RALC referrals, MDT meetings, and timely treatment commencements. In fact, shorter wait times for surgery was observed during the pandemic indicating a potential reduction in elective surgeries, or cancer-related surgeries being prioritised. Furthermore, median PFS and OS of patients diagnosed with lung cancer at CUH-RALC was similar between the periods investigated indicating no impact of the COVID-19 pandemic on the short-term cancer outcome for patients, within the study time frame. This demonstrates the successful robust, reactive, risk mitigating strategies that were implemented early by thoracic leadership to prioritise continued CT cancer detection, which prevented the potential negative consequence of the pandemic, particularly with respect to cancer-specific mortality.

The development of Rapid Access Clinic by the NCCP in 2009 has been critical for timely diagnosis and access to hospital-based treatments for cancer patients in Ireland. The results of this study show how crucial these services are particularly during a national health crisis, like the COVID-19 pandemic. However, our findings may not reflect the performance of other RALC centres in Ireland. Our analysis was limited to the two years surrounding the COVID-19 pandemic and the data on year-to year variability along with patient category of GP vs. ED/inpatient referrals were not analysed in this study. Furthermore, our follow-up period for PFS and OS was limited to 7 months; therefore, we were unable to determine the effects of the pandemic on lung cancer outcome in the long term. Although similar trends are seen in the number of referrals to the RALC centres in Cork, Limerick, and Beaumont, other confounding factors such as staffing, access to diagnostics, variations in COVID-19 local protocols, and total number of annual referrals might influence variations seen among centres during the pandemic. Ultimately, the prevention, screening, and survivorship programmes developed by the NCCP play an important role in reducing the burden of cancer in Ireland. As with this study, the quality and performance of these programmes must be routinely measured to ensure that they meet their respective recommendations and KPIs.

Strengths of this study include large patient cohort, robust statistical analysis when comparing periods, and inclusion of PFS and OS with a follow-up period. Limitations of the analysis include the size and retrospective design of the study, absence of data from other RALC centres in Ireland, lack of data for the annual period of the pandemic post this analysis, short follow-up period, and confounding factors like change in staff. Furthermore, separate analysis on GP and emergency department referrals to RALC would help clarify the impact of COVID-19 on the primary care in Ireland.

In conclusion, we have presented reassuring data surrounding the performance of our rapid access lung cancer clinic during the COVID-19 pandemic. However, we must continue to strive to improve our compliance with the NCCP KPIs by increasing efficacy of the RALC service and pathways which will ultimately improve survival outcomes over the next decade. Longer follow-up data and additional investigations would provide further insight into the full impact of the COVID-19 pandemic on outcomes of patients at risk of and diagnosed with lung cancer in Ireland and indeed globally. A national evaluation will help determine the full impact of this pandemic on lung cancer diagnosis, management, and long-term outcomes.
